# Evaluation of Short Versus Long Course of Tobramycin Combined with Piperacillin/Tazobactam Against Antibiotic-Resistant *Pseudomonas aeruginosa* in a Hollow Fibre Infection Model

**DOI:** 10.3390/antibiotics15070685

**Published:** 2026-07-13

**Authors:** Yalew M. Wale, Jason A. Roberts, Hayoung Won, Sheethal Reghu, Dusan Marjanovic, Getnet M. Assefa, Budi Permana, Brian Ford, Fekade B. Sime

**Affiliations:** 1 Frazer Institute, Faculty of Health, Medicine and Behavioral Sciences, The University of Queensland, Brisbane 4029, Australia; 2 Department of Pharmacy, College of Health Sciences, Debre Markos University, Debre Markos P.O. Box 269, Ethiopia; 3 Departments of Pharmacy and Intensive Care Medicine, Royal Brisbane and Women’s Hospital, Brisbane 4029, Australia; 4 Division of Anaesthesiology Critical Care Emergency and Pain Medicine, Nîmes University Hospital, University of Montpellier, UR UM 103, 34090 Nimes, France; 5 Herston Infectious Diseases Institute (HeIDI), Metro North Health, Brisbane 4006, Australia; 6 Department of Pharmacy, College of Medicine and Health Sciences, Wollo University, Dessie P.O. Box 1145, Ethiopia; 7 Institute for Molecular Bioscience, The University of Queensland, Brisbane 4029, Australia; 8 School of Pharmacy and Pharmaceutical Sciences, Faculty of Health, Medicine and Behavioral Sciences, The University of Queensland, Brisbane 4029, Australia

**Keywords:** aminoglycoside therapy, tobramycin, piperacillin/tazobactam, *Pseudomonas aeruginosa*, hollow fibre infection model

## Abstract

Aminoglycosides are commonly partnered with β-lactam antibiotics for empirical treatment of severe Gram-negative bacterial infections, including those caused by *Pseudomonas aeruginosa*. However, the optimal duration of aminoglycoside therapy when co-administered with β-lactam antibiotics remains poorly defined. This study compared the antibacterial efficacy of short (one-day, three-day) versus long course (seven-day) of tobramycin therapy, co-administered with piperacillin/tazobactam, against piperacillin/tazobactam-resistant clinical isolates of *P. aeruginosa* in a hollow fibre infection model (HFIM). A broth microdilution method was used to determine the minimum inhibitory concentration (MIC) of the antibiotics. The HFIM experiments were performed using two piperacillin/tazobactam-resistant clinical isolates, CTAP-32 and CTAP-72 (both with piperacillin/tazobactam MICs > 128 mg/L). The clinical dose of tobramycin (10 mg/kg/day IV) was simulated for one, three, and seven days, each in combination with piperacillin/tazobactam (4.5 g every six hours infused over 30 min). In the HFIM, the one-day, three-day, and seven-day courses of tobramycin administered with piperacillin/tazobactam resulted in comparable bacterial kill with ~3log_10_ CFU/mL bacterial density reduction within the first 8 h of treatment against the CTAP-32 bacterial isolate. Similarly, these three combination regimens resulted in equivalent bacterial killing effects against the CTAP-72 isolate with a ~5log_10_ CFU/mL bacterial reduction during the initial treatment period. However, despite this early decline, comparable bacterial regrowth was observed thereafter with the short versus the long course of tobramycin co-administered with piperacillin/tazobactam. In conclusion, short-course and long-course tobramycin therapy, when combined with piperacillin/tazobactam, achieved comparable antibacterial efficacy against *P. aeruginosa* isolates in the HFIM. However, these findings warrant further validation in clinical studies.

## 1. Introduction

Infections caused by Gram-negative bacteria pose a significant global threat to human health. These infections are associated with high morbidity and mortality rates and are notoriously difficult to treat [1,2]. The challenges in managing Gram-negative bacterial infections stem largely from their ability to develop resistance to multiple antibiotics [3,4]. Antibiotic resistance is particularly common among the ESKAPE pathogens (*Enterococcus faecium*, *Staphylococcus aureus*, *Klebsiella pneumoniae*, *Acinetobacter baumannii*, *Pseudomonas aeruginosa*, and *Enterobacter species*) [5,6]. Among these, *P. aeruginosa* is a leading cause of infections, including respiratory infections, bacteraemia, urinary tract infections, and intra-abdominal infections [7,8,9,10]. It is estimated that *P. aeruginosa* accounts for 7–20% of nosocomial infections in most hospitals, resulting in prolonged admissions [10,11].

Initial empiric therapy of severe infections caused by Gram-negative bacteria such as *P. aeruginosa* often involves treatment with the combinations of two or more antibiotics known to have synergistic antibacterial effects [12,13]. Among the commonly used combination regimens are those of aminoglycosides and β-lactam antibiotics, which exhibit synergistic activity [14,15]. However, prolonged use of aminoglycosides has been associated with the development of adverse effects such as nephrotoxicity and ototoxicity [16]. To mitigate these risks, physicians are increasingly adopting short-course aminoglycoside therapies (less than three days) in combination with β-lactam antibiotics for empiric treatment of severe Gram-negative bacterial infections [17]. This approach is recommended by some treatment guidelines, including the Australian guidelines, to reduce aminoglycoside-related toxicities [18]. Despite these recommendations, there is considerable variability in the duration of aminoglycoside therapy used in combination with β-lactam antibiotics [19]. Optimising the duration of therapy is crucial to achieve effective bacterial eradication while minimising adverse effects, potentially resulting in shorter hospital stays and reduced healthcare costs [16,20].

The optimal duration of aminoglycoside therapy when used in combination with β-lactam antibiotics for empiric treatment of severe infections caused by antibiotic-resistant Gram-negative bacteria remains a subject of debate. Existing evidence presents conflicting findings regarding the efficacy of short- versus long-course aminoglycoside therapy when used with β-lactam antibiotics. Some studies have suggested that prolonged aminoglycoside therapy administered with the full course of β-lactam antibiotics resulted in superior antibacterial efficacy [21]. In contrast, other cohort studies indicate that short-course therapy is adequate to manage these Gram-negative bacterial infections [22,23]. However, the empiric treatment efficacy of short- versus long-course aminoglycoside therapy against severe Gram-negative bacterial infections, when combined with β-lactam antibiotics, remains insufficiently supported by comparative clinical or controlled dynamic in vitro studies. Therefore, the purpose of this study was to compare short versus long courses of tobramycin therapy combined with piperacillin/tazobactam against piperacillin/tazobactam-resistant *P. aeruginosa* clinical isolates in a hollow fibre infection model (HFIM). The HFIM is widely regarded as a gold-standard tool for simulating the human antibiotic pharmacokinetics profiles, providing critical insights about bacteriological responses and informing clinical decisions [24,25]. The use of hollow fibre infection models also avoids the ethical issues required for conducting comparative clinical studies [24].

## 2. Results

### 2.1. In Vitro Susceptbility of the Bacterial Isolates

The MICs of tobramycin and PIPTAZ to the *P. aeruginosa* isolates is depicted in Table 1. The bacterial isolates CTAP-32 and CTAP-72 were resistant to both tobramycin (MIC > 2 mg/L) and PIPTAZ (MIC > 16 mg/L) in accordance with EUCAST clinical susceptibility breakpoints.

### 2.2. Static Concentration Time-Kill Study

**Figure 1:**
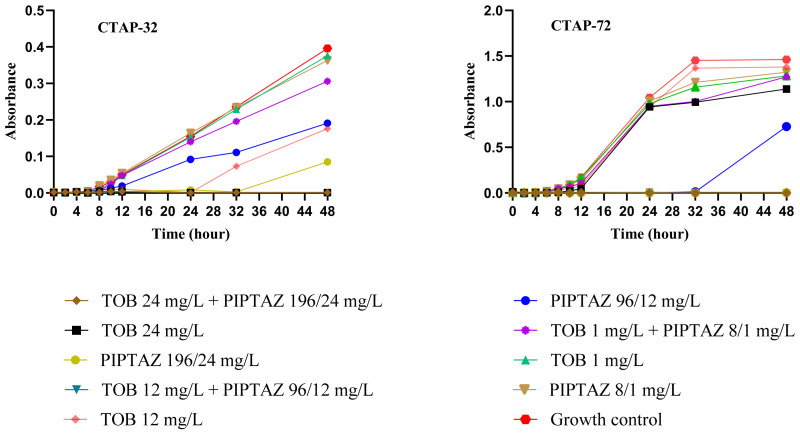
The optical density (at 620 nm) of *P. aeruginosa* isolates (CTAP-32 and CTAP-72) exposed to different static concentrations of tobramycin and PIPTAZ in monotherapy and combination therapy. TOB, tobramycin; PIPTAZ, Piperacillin/tazobactam.

The combined use of medium concentrations of tobramycin (12 mg/L) and PIPTAZ (96/12 mg/L) demonstrated enhanced bacterial reduction activity compared to each antibiotic used alone against both CTAP-32 and CTAP-72 Pseudomonas isolates. Additionally, the combined use of low concentrations of tobramycin (1 mg/L) and PIPTAZ (8/1 mg/L) showed greater bacterial reduction effects compared to each antibiotic used alone at the same concentrations against the CTAP-32 isolate (Figure 1).

### 2.3. Bacterial Population Profile Analysis

After 48 h of incubation at 37 °C, the bacterial density of both isolates on antibiotic-containing agar appeared to be concentration-dependent. Both CTAP-32 and CTAP-72 grew on both tobramycin and PIPTAZ containing agar at all the concentrations tested. However, CTAP-32 did not grow on tobramycin-containing agar at a concentration of 64 mg/L (Table 2).

### 2.4. Hollow Fibre Infection Model

**Figure 2:**
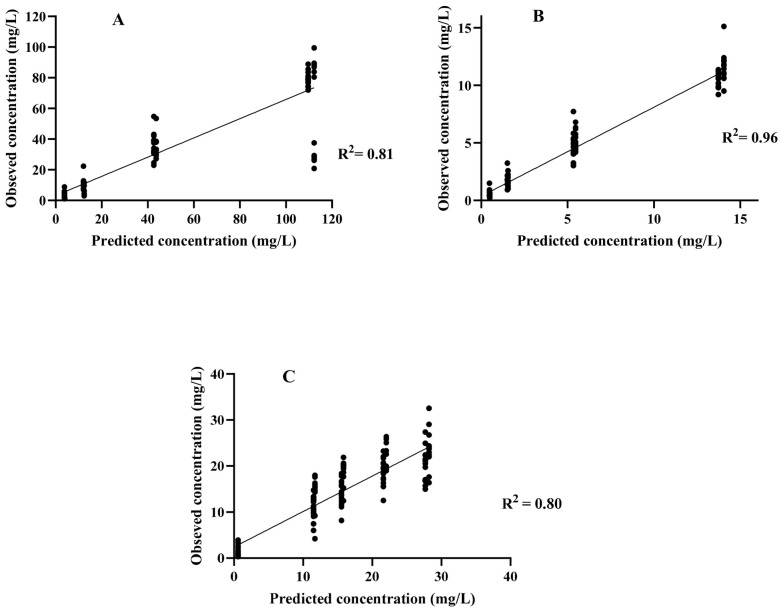
The predicted versus observed concentration-time plots of piperacillin (**A**), tazobactam (**B**), and tobramycin (**C**) in the hollow fibre infection model.

The concentration-time profiles of the test antibiotics were adequately simulated in the HFIM (Figure 2). The observed concentrations of PIPTAZ and tobramycin are within the acceptable range of target antibiotic exposure throughout the study period.

**Figure 3:**
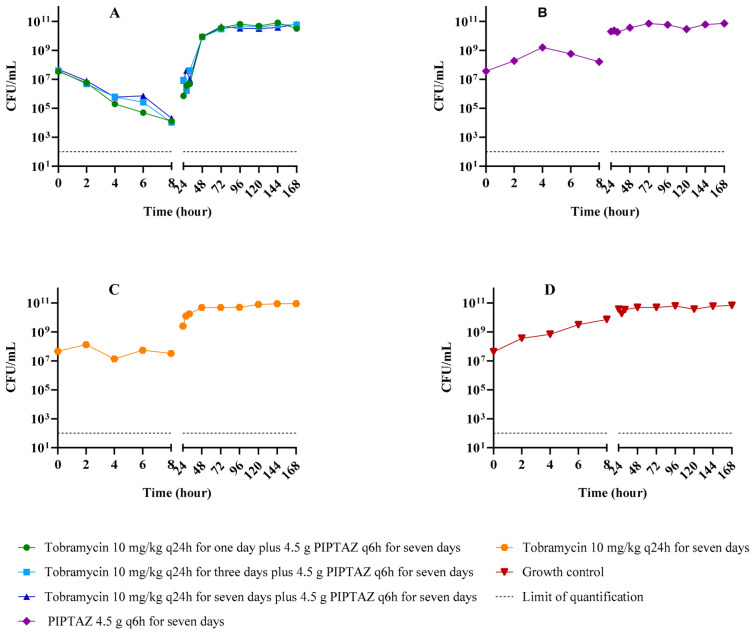
Time-course bacterial densities of *P. aeruginosa* isolate CTAP-32 in hollow fibre infection model following treatment with (**A**) tobramycin plus PIPTAZ, (**B**) PIPTAZ alone, (**C**) tobramycin alone, and (**D**) growth control. CFU, colony forming units; q24h, every 24 h; q6h, every 6 h.

**Figure 4:**
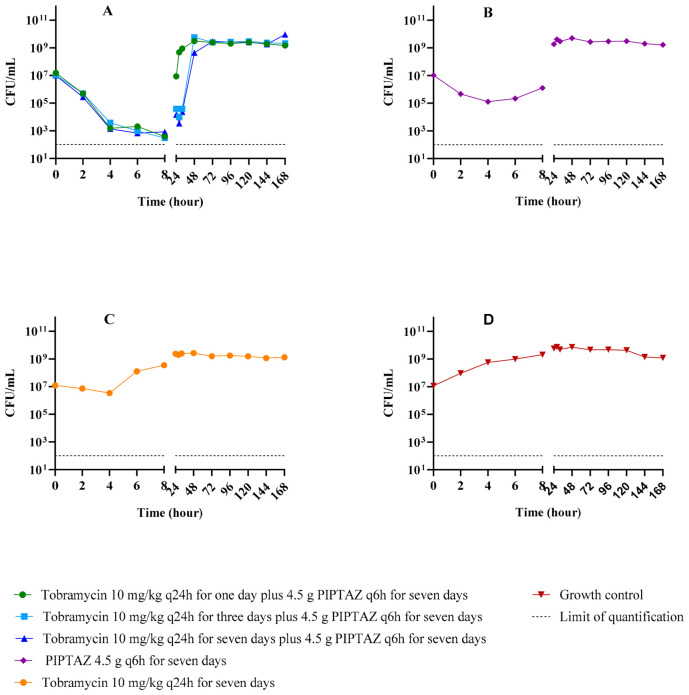
Time-course bacterial densities of *P. aeruginosa* isolate CTAP-72 in hollow fibre infection model following treatment with (**A**) tobramycin plus PIPTAZ, (**B**) PIPTAZ alone, (**C**) tobramycin alone, and (**D**) growth control. CFU, colony forming units; q24h, every 24 h; q6h, every 6 h.

The time-course changes in the bacterial density following treatment with various regimens against the two study isolates are depicted in Figure 3 and Figure 4. The HFIM experiment revealed that short-course tobramycin therapy, when combined with PIPTAZ, resulted in a bacterial load reduction comparable to that observed with long-course therapy against the tested *P. aeruginosa* isolates. Specifically, the administration of tobramycin for one day, three days, and seven days in combination with PIPTAZ achieved an approximate 3log_10_ CFU/mL reduction in bacterial density within the first 8 h of treatment against the CTAP-32 *P. aeruginosa* isolate (Figure 3A). A similar trend was observed for the CTAP-72 isolate, where all three tobramycin combination regimens resulted in a 5log_10_ CFU/mL bacterial reduction within the same timeframe (Figure 4A). This initial bacterial density reduction was consistent across all three combination regimens, irrespective of the duration of tobramycin administered with PIPTAZ. However, following this early bacterial load reduction, regrowth of the two *P. aeruginosa* isolates was noted after the eighth hour of antibiotic exposure.

The regrowth patterns of the *P. aeruginosa* isolates were similar across all three combination regimens of tobramycin as shown in Figure 3A and Figure 4A, showing no differences based on the duration of tobramycin administration. After 24 h, neither the combination regimens nor monotherapy treatments were able to control bacterial resurgence, and this trend persisted throughout the seven-day experiment.

The three combination regimens of tobramycin and PIPTAZ demonstrated a synergistic antibacterial effect against CTAP-32, reducing bacterial load by more than 2log_10_ CFU/mL compared with the active monotherapy (tobramycin alone) during the initial phase of antibiotic therapy period. Similarly, in the case of CTAP-72, the three combination regimens of tobramycin and PIPTAZ resulted in a synergistic bactericidal effect, reducing the bacterial load by more than 2log_10_ CFU/mL compared to the active monotherapy (PIPTAZ alone) during the first day of initiating the antibiotic therapy (Figure 3 and Figure 4).

### 2.5. Whole Genome Sequencing and Bioinformatic Analysis

The whole genome sequencing revealed that both CTAP-32 and CTAP-72 *P. aeruginosa* isolates conferred genes resistant to various antibiotics, including aminoglycosides, β-lactams, fosfomycin, chloramphenicol, and quinolones. Additionally, the CTAP-72 isolate presented genes resistant to sulphonamides (Table 3).

The *P. aeruginosa* isolates used in the HFIM did not show any change in the presence of the antibiotic-resistant genes over the 168 h of initiating antibiotic therapy (Table 3). The distribution of these antibiotic-resistant genes was similar across the treatment arms over the experiment period (Table 3).

## 3. Discussion

Aminoglycosides are administered for different durations when combined with β-lactam antibiotics to treat Gram-negative bacterial infections. Optimising the duration of aminoglycoside therapy in combination with β-lactam antibiotics is crucial to minimise aminoglycoside-associated toxicities while ensuring effective bacterial killing. This study compared the antibacterial effects of the short versus the long course of tobramycin administered with PIPTAZ against antibiotic-resistant *P. aeruginosa* clinical isolates in an HFIM. Two major findings were observed in this study. First, short-course (one-day and three-day) tobramycin therapy in combination with PIPTAZ demonstrated a bacterial reduction effect comparable to that of the long course (seven-day) combination regimen, achieving a 3–5log_10_ CFU/mL reduction in bacterial density during the early treatment period. Second, irrespective of the duration of tobramycin therapy, the three tobramycin–PIPTAZ combination regimens demonstrated synergistic antibacterial effect, with a bacterial reduction exceeding 2log_10_ CFU/mL relative to their monotherapy regimens during the initial phase of the antibiotic therapy.

The time-course bacterial count profiles across the three tobramycin–PIPTAZ combination regimens exhibited similar trends over the seven-day experimental period against *P. aeruginosa* clinical isolates. Regardless of whether tobramycin was administered for one, three, or seven days in combination with PIPTAZ, the antibacterial effect remained comparable during the first day of antibiotic therapy, demonstrating a consistent bacterial killing pattern across these regimens. The equivalent antibacterial effects of the short versus the long course of the tobramycin combined with PIPTAZ support the growing clinical preference for limiting aminoglycoside exposure while combined with β-lactam antibiotics. Current clinical guidelines [18] recommend administering aminoglycosides for short durations (typically less than three days) while maintaining a full course of β-lactam antibiotics for the empirical treatment of Gram-negative bacterial infections. This strategy is driven by the need to optimise bacterial eradication while minimising the risks associated with prolonged aminoglycoside use. By limiting aminoglycoside exposure to the shortest effective duration, clinicians can reduce the likelihood of adverse effects while achieving therapeutic success. The effectiveness of administering aminoglycosides for a short duration with β-lactam antibiotics against Gram-negative bacteria observed in this study has also been supported by some other clinical studies [22,26]. The comparable antibacterial effects observed between short and long courses of tobramycin combination regimens in this study could be attributed, at least in part, to the prolonged post-antibiotic effect of aminoglycosides [18], which sustain bacterial killing even after short treatment durations. Therefore, this HFIM study provided experimental evidence for the existing antimicrobial stewardship principles and current clinical practice favouring a short course of tobramycin as part of combination therapy to minimise aminoglycoside-associated toxicities. Importantly, this HFIM evidence provides clinically relevant insights that are often difficult to obtain through clinical studies alone.

The enhanced bacterial killing observed with the administration of tobramycin in combination with PIPTAZ, compared to their respective monotherapy regimens, is likely due to their synergistic pharmacodynamic interactions (killing more than 2log CFU/mL bacterial load over the active monotherapy) [27]. One plausible mechanism of synergy is that piperacillin disrupts the bacterial cell wall, facilitating increased penetration of tobramycin into the bacterial cell. Conversely, the outer-membrane-disrupting effect of tobramycin on Gram-negative bacteria may enhance the uptake of piperacillin to the target site, further potentiating its antibacterial activity [28,29]. These synergistic effects of the combination of tobramycin and PIPTAZ were also supported by the findings observed in static concentration time-kill experiments, although the optical density measurements do not distinguish between the vial and non-viable bacterial cells [30].

The tobramycin–PIPTAZ combination regimens reduced the bacterial population to approximately 2log_10_ CFU/mL against CTAP-72 and to ~4log_10_ CFU/mL against CTAP-32 in the HFIM. This paramount bacterial reduction, particularly against the CTAP-72 isolate, suggests that these regimens could potentially lead to complete treatment of the infection in immunocompetent patients, as the host immune system could effectively eliminate the residual bacterial population [31]. A previous mouse model study indicated that a pharmacodynamic bacterial load reduction to ≤5log_10_ CFU/g of tissue is needed at the initial antibiotic treatment period to observe the optimal bacterial killing action of granulocytes [32]. This antibacterial effect is necessary to decrease the saturation of white blood cells and enhance their bacterial killing actions [32]. Therefore, the initial bacterial load reduction effect achieved in the present study with the short duration of aminoglycoside-based combination therapy may result in clinical cure in immunocompetent patients infected with *P. aeruginosa*.

The initial antibacterial effects of the combination regimens were not consistent against the two *P. aeruginosa* isolates selected for this study. Their antibacterial effect was greater against the fast-growing isolate CTAP-72 (5log_10_ CFU/mL load reduction) compared to the slow-growing isolate CTAP-32 (3log_10_ CFU/mL load reduction). The observed difference in bacterial killing effects between the two *P. aeruginosa* isolates in the combination regimens could be attributed to the low efficacy of the bactericidal antibiotics against the slow-growing bacteria [33]. Additionally, this difference could be related to the difference in type and the distribution of the antibiotic resistance genes presented across the selected *P. aeruginosa* clinical isolates. However, both *P. aeruginosa* isolates showed regrowth following the initial bacterial load reduction within the first 24 h of antibiotic therapy with the combination of tobramycin and PIPTAZ. None of the regimens evaluated in the HFIM were effective in controlling the regrowth of the bacterial isolates.

The regrowth of the *P. aeruginosa* isolates observed in this study may be attributed to several factors. Firstly, the bacterial culture might contain multidrug-resistant subpopulations, which could repopulate during the treatment period due to antibiotic selection pressure [34]. Indeed, this is supported by the population profile analysis of the study isolates, which confirmed the presence of heteroresistance. The presence of heteroresistant populations of *P. aeruginosa* for PIPTAZ was also reported by a previous study [35]. The bacterial regrowth could result from the overexpression of pre-existing antibiotic resistance genes under stress to antibiotic treatment exposure. For example, the resistance of some *P. aeruginosa* isolates to PIPTAZ has been associated with the expression of ampC, *blaOXA-50*, *blaOXA-488*, *MexA* and *mexE* antibiotic resistance genes [36,37,38]. Similarly, for tobramycin, the presentation of *fusA1_Y552C* and *mexX* genes has been implicated as a potential mechanism of resistance in *P. aeruginosa* isolates [39,40,41]. Congruent to these reports, CTAP-32 consistently harboured *fusA1_Y552C*, *blaOXA-50*, *mexA*, *mexE*, and *mexX* while CTAP-72 carried *blaOXA-488*, *mexA*, *mexE*, and *mexX* both in baseline and end of treatment assessments. Therefore, the failure of the combination regimens to suppress the regrowth of the bacteria in this study could be attributable to the amplification of these antibiotic resistance genes upon exposure to treatment regimens. Secondly, the antibiotic exposure could drive the development of new adaptive mechanisms such as biofilm formation in the HFIM cartridge, contributing to the regrowth of the bacteria observed in this study [42].

This study has some limitations. First, the HFIM used does not replicate the human immune system, which plays a crucial role in bacterial clearance among immunocompetent patients. However, this model remains valuable for simulating antibiotic exposure–response relationships in immunocompromised individuals [43]. Second, the study was performed using a single broth flow rate simulating normal renal function (creatinine clearance of 100 mL/min). Consequently, the findings may not fully reflect antibiotic pharmacokinetics in patients with altered renal function, such as those with augmented renal clearance or impaired renal function, where drug exposure can differ significantly. Third, the study did not undertake the functional studies, including transcriptomic analysis and biofilm assessment, to address the mechanism underlying bacterial regrowth observed in the HFIM study. Lastly, the study was conducted using a limited number of *P. aeruginosa* isolates due to the resource-intensive nature of HFIM experiments, which may limit the broader generalisability of our findings to treat infections caused by other Gram-negative bacteria.

## 4. Methods

### 4.1. Antibiotic Preparations

Analytical reference standards of piperacillin sodium (Sigma-Aldrich, St. Louis, MO, USA), tazobactam sodium (Fluorochem Ltd., Glossop, UK), and tobramycin (Tokyo Chemical Industry Ltd., Tokyo, Japan) were used for minimum inhibitory concentration (MIC), static concentration time-kill, and bacterial population profiling tests. Clinical formulations of piperacillin/tazobactam (PIPTAZ) (AFT Pharmaceuticals Ltd., Auckland, New Zealand) and tobramycin (Viatris Ltd., Auckland, New Zealand) were used for dosing simulations in the HFIM.

The simulating doses of PIPTAZ and tobramycin were prepared at a specific stock concentration for use in the HFIM. These stock solutions were aliquoted and stored at −80 °C until needed. Each aliquot was thawed and reconstituted with cation-adjusted Mueller–Hinton broth (CAMHB) (BD, Sparks, MD, USA) immediately before administration. The antibiotics were administered using syringe pumps.

### 4.2. Bacterial Inoculum Preparations

Clinical isolates of *P. aeruginosa* (CTAP-32 and CTAP-72) were obtained from the Antimicrobial Optimization Group’s bacterial stocks at the University of Queensland, Centre for Clinical Research (UQCCR). Each bacterial inoculum was prepared by colony suspension method [44]. First, bacterial inoculums of these isolates equivalent to 0.5 McFarland standard were prepared from freshly grown bacterial culture with colony suspension method. This bacterial density was diluted to 1 × 107 colony forming units per millilitre (CFU/mL) and 1 × 106 CFU/mL in phosphate-buffered saline (PBS), to conduct the static concentration time-kill and MIC determination experiments, respectively.

The 0.5 McFarland standard equivalent bacterial inoculum was further diluted in PBS to 1 × 102 CFU/mL to conduct the bacterial growth profile determination experiments. For the HFIM experiments, the diluted bacterial inoculum equivalent to ~1 × 107 CFU/mL was used. The quantitative load of each inoculum sample was confirmed through agar plating and colony counting techniques.

### 4.3. Minimum Inhibitory Concentration

The MIC of each antibiotic was determined using the broth microdilution method, in accordance with the European Committee on Antimicrobial Susceptibility Testing (EUCAST) guidelines [44]. Stock solutions of each antibiotic were prepared and then serially diluted two-fold in CAMHB.

Aliquots of 100 µL from each antibiotic dilution were dispensed into round-bottom microtiter plate wells with additional wells included as sterility and growth controls. For the PIPTAZ MIC assay, the concentration of tazobactam in each serial dilution well was fixed at 4 mg/L. A bacterial suspension prepared in CAMHB was added to each well, except the sterility control wells, to achieve a final bacterial concentration of 5 × 105 CFU/mL. The plate was incubated at 37 °C for 20 h. The MIC was defined as the lowest modal concentration of the antibiotic that completely inhibited the visible bacterial growth. Each MIC test was conducted in quadruplicate.

### 4.4. Static Concentration Time-Kill Study

A static time-kill study was performed to evaluate the effects of varying concentrations of tobramycin (1, 12, 24 mg/L) and PIPTAZ (8/1, 96/12, 196/24 mg/L), both individually and in combination, against *P. aeruginosa* clinical isolates. For this purpose, the appropriate volume of either a single antibiotic or the antibiotic combination stock solution was mixed with the bacterial inoculum and incubated at 37 °C for 24 h in a shaking incubator set at 100 RPM.

After 24 h incubation, the bacterial suspension was centrifuged and washed carefully. Thereafter, fresh CAMHB and the required volume of each antibiotic stock solution were added back to the test tubes to replace the removed broth and antibiotics. The bacterial pellets were resuspended and incubated further at 37 °C for an additional 24 h.

Samples of the bacterial suspension were collected from each tube at defined time points—0, 2, 4, 6, 8, 12, 24, 32, and 48 h—to evaluate the bacterial killing activity of the antibiotic treatments. The bacterial growth kinetics of each sample were assessed by measuring its optical density at 620 nm relative to the negative control. This experiment was conducted without replicates.

### 4.5. Bacterial Population Profile Analysis

A bacterial population profile analysis study was conducted by enumerating the growth of *P. aeruginosa* isolates on Mueller–Hinton agar (MHA) plates containing two-fold serial dilutions of the test antibiotics, starting from the clinical MIC breakpoints as per the EUCAST guidelines [45]. For PIPTAZ, MHA plates were prepared with concentrations of 16/4, 32/4, 64/4, and 128/4 mg/L. For tobramycin, MHA plates with concentrations of 2, 4, 8, 16, 32, and 64 mg/L were prepared.

The bacterial inoculum was prepared at 37 °C for 24 h, of which 100 µL samples of the inoculum were serially diluted in PBS, and plated onto the prepared MHA plates containing the various concentrations of the test antibiotics. Bacterial density was assessed by counting bacterial colonies grown on the agar plates.

### 4.6. Hollow Fibre Infection Model

The HFIM experiment was conducted using a cellulosic cartridge (C3008, FiberCell Systems, Inc., Frederick, MD, USA) against the CTAP-32 isolate and a polysulfone cartridge (C2011, FiberCell Systems, Inc., Frederick, MD, USA) against the CTAP-72 isolate. The bacterial inoculum of each isolate was injected into the extra-capillary space (ECS) of the cartridges. The duet pump was set at a high flow rate (~80 mL/min) to achieve a rapid exchange of antibiotics and nutrients between the central compartment and the ECS.

To replicate the differing clearances of tobramycin and PIPTAZ, a supplementary system was integrated in the HFIM circuit, based on the Blaser description [46]. The pharmacokinetics of tobramycin and PIPTAZ within the HFIM were simulated using population pharmacokinetics described by Xuan et al. [47] and Chen et al. [48], respectively. The simulated half-lives of tobramycin and PIPTAZ were 4.2 h and 1.1 h, respectively, assuming an 80 kg body weight and a creatinine clearance of 100 mL/min. The volume of the central compartment was 150 mL. Hence, the corresponding flow rates of tobramycin and PIPTAZ into the central compartment were set at 0.41 mL/min and 1.58 mL/min, respectively.

Six independent HFIM circuit systems were set up in duplicate to model the clinical antibiotic therapy for the following regimens:

- Tobramycin 10 mg/kg q24h for one day plus 4.5 g PIPTAZ q6h for seven days;
- Tobramycin 10 mg/kg q24h for three days plus 4.5 g PIPTAZ q6h for seven days;
- Tobramycin 10 mg/kg q24h for seven days plus 4.5 g PIPTAZ q6h for seven days;
- PIPTAZ monotherapy 4.5 g q6h for seven days;
- Tobramycin monotherapy 10 mg/kg q24h for seven days;
- Growth control.

These simulated doses of the antibiotics were administered by intermittent infusion over 30 min using syringe pumps.

Bacterial samples were collected at 0, 2, 6, 8, 24, 28, 32, 48, 72, 96, 120, 144, and 168 h. Antibiotic-containing samples were washed with sterile PBS to avoid drug carryover, and the bacterial pellets were resuspended using a vortex mixer. Each sample was serially diluted 10-fold, and 100 µL of the diluted sample was plated on antibiotic-free MHA. Bacterial colonies were enumerated after 24 h of incubation at 37 °C for the CTAP-72 isolate, and after 48 h for CTAP-32 (the slowly growing isolate), to determine the total bacterial density in each treatment arm. For pharmacokinetic analysis, fluid samples were taken from the central compartment at 0.5, 2, 4, 5.83, 23.83, 48.5, 50, 52, 53.83, 71.83, 96.5, 98, 100, 101.83, and 119.83 h of the treatment period.

### 4.7. Antibiotic Assay

Piperacillin/tazobactam and tobramycin concentrations in CAMHB were measured by an ultra-high performance liquid chromatography system coupled with a tandem mass spectrometry (UHPLC-MS/MS) method on a Nexera UHPLC connected to a 8030+ triple quadrupole mass spectrometer (Shimadzu, Kyoto, Japan).

The test samples were assayed in batches alongside calibrators and quality control (QC) samples, and the results were subject to batch acceptance criteria [49]. The precision and accuracy of the assays were within acceptable limits. The precision for piperacillin was within 7.7% and accuracy was within 6.5%, based on QC concentrations of 1.5, 15, 50, and 400 mg/L. For tazobactam, precision was within 13.7% and accuracy was within 3.5%, using QC concentrations of 0.1875, 1.875, 6.25, and 50 mg/L. The tobramycin assay method demonstrated precision within 8.8% and accuracy within 3.8%, based on the QC concentrations of 0.6, 1.5, 15, and 40 mg/L.

### 4.8. DNA Extraction, Whole Genome Sequencing, and Bioinformatic Analysis

Whole genome sequencing (WGS) was conducted on an Illumina Nextseq2000 with XLEAP-SBS chemistry, using a 300-cycle paired end P3 flow cell, according to the manufacturer’s protocol. The raw Illumina sequencing reads were processed and analysed using a custom, in-house microbial genomic analysis pipeline, SnapperRocks [50]. The detailed procedure for WGS is described under the Supplementary Data.

## 5. Conclusions

The administration of tobramycin for a short (one day or three days) versus long course (seven days) with PIPTAZ resulted in comparable bacteriological outcomes against PIPTAZ-resistant *P. aeruginosa* in the HFIM. This suggests that a short course of tobramycin combined with PIPTAZ maybe adequate for the empirical treatment of severe infections caused by PIPTAZ-resistant *P. aeruginosa*. However, further clinical studies are necessary to validate these findings in clinical settings.

## Figures and Tables

**Table 1:** Susceptibility of *P. aeruginosa* isolates to tobramycin and PIPTAZ.

Isolates	MIC Results			
	Tobramycin		PIPTAZ	
	MIC (mg/L)	Susceptibility	MIC (mg/L)	Susceptibility
CTAP-32	16	R	>128/4	R
CTAP-72	64	R	>128/4	R

MIC, minimum inhibitory concentration; R, resistant.

**Table 2:** Bacterial growth profile of *P. aeruginosa* isolates on agar with or without different tobramycin or PIPTAZ concentrations.

Antibiotic Concentrations in Agar		Bacterial Density (CFU/mL)	
Antibiotic	Concentration (mg/L)	CTAP-32	CTAP-72
Tobramycin	2	3.3 × 10^8^	3 × 10^8^
	4	3.1 × 10^8^	3.2 × 10^8^
	8	9.4 × 10^7^	2.26 × 10^8^
	16	6.7 × 10^4^	1.04 × 10^7^
	32	4.5 × 10^2^	5.2 × 10^4^
	64	0.00	1.16 × 10^4^
PIPTAZ	16/4	9.6 × 10^7^	1.47 × 10^5^
	32/4	1.18 × 10^8^	1.35 × 10^5^
	64/4	9.7 × 10^7^	9.6 × 10^4^
	128/4	3.3 × 10^7^	2.7 × 10^4^
Antibiotic-free agar	0	3.2 × 10^8^	2.6 × 10^8^

CFU, colony forming units.

**Table 3:** The whole genome sequence of the *P. aeruginosa* isolates used in the HFIM experiment.

Isolate	Treatment Arm	Antibiotic-Resistant Genes	MLSTs
CTAP-32	Prior to antibiotic exposure	*aph(3′)-IIb*, *fusA1_Y552C*, *blaOXA-50*, *blaPDC-97*, *ftsI_R504C*, *oprD_V359L*, *mexA*, *mexE*, *mexX*, *fosA*, *catB7*, *gyrA_T83A*	782
	A, B, and C	*aph(3′)-IIb*, *fusA1_Y552C*, *blaOXA-50*, *blaPDC-97*, *ftsI_R504C*, *oprD_V359L*, *mexA*, *mexE*, *mexX*, *fosA*, *catB7*, *gyrA_T83A*	
CTAP-72	Prior to antibiotic exposure	*aph(3′)-IIb*, *aadA6*, *blaOXA-488*, *blaIMP-1*, *blaPDC-35*, *mexA*, *mexE*, *mexX*, *crpP*, *fosA*, *catB7*, *gyrA_T83I*, *sul1*	235
	A, B, C, and D	*aph(3′)-IIb*, *aadA6*, *blaOXA-488*, *blaIMP-1*, *blaPDC-35*, *mexA*, *mexE*, *mexX*, *crpP*, *fosA*, *catB7*, *gyrA_T83I*, *sul1*	

HFIM, hollow fibre infection model; MLSTs, multi-locus sequence types; A, tobramycin 10 mg single dose plus PIPTAZ 4.5 g q6h for 7 days; B, tobramycin 10 mg q24h 3 days plus PIPTAZ 4.5 g q6h for 7 days; C, tobramycin 10 mg q24h 7 days PIPTAZ 4.5 g q6h for 7 days; D, PIPTAZ 4.5 g q6h for 7 days.

## Data Availability

The whole genome sequence read data have been deposited in NCBI Sequence Read Archive with the BioProject number PRJNA1465934 and accession number SAMN59841686, SAMN59841687, SAMN59841688, SAMN59841689, SAMN59841690, SAMN59841691, SAMN59841692, SAMN59841694, and SAMN59841695.

## Supplementary Materials

The following supporting information can be downloaded at https://www.mdpi.com/article/10.3390/antibiotics15070685/s1, Antibiotic assay; DNA extraction, whole genome sequencing, and bioinformatic analysis; References mentioned in the supplementary material have been cited in the main text [31,32,51,52,53,54,55].
