# Supplementary material for: Evaluation of Short Versus Long Course of Tobramycin Combined with Piperacillin/Tazobactam Against Antibiotic-Resistant Pseudomonas aeruginosa in a Hollow Fibre Infection Model
Source: Antibiotics (Basel). 2026 Jul 13;15(7):685. doi: 10.3390/antibiotics15070685 (PMC13405948; doi:10.3390/antibiotics15070685)
Supplement: Supplementary file 1 [file antibiotics-15-00685-s001.zip › antibiotics-4391795-supplementary.pdf]

Supplementary material

Antibiotic Assay

Piperacillin/tazobactam and tobramycin concentrations in CAMHB were measured by an ultra-high performance liquid chromatography system coupled with tandem mass spectrometry (UHPLC-MS/MS) method on a Nexera UHPLC connected to a 8030+ triple quadrupole mass spectrometer (Shimadzu, Kyoto, Japan). Calibration standards and quality control (QC) samples were prepared in water and drug-free CAMHB respectively and analysed along with the test samples.

For piperacillin/tazobactam, 20 μL aliquots of standards were mixed with 20 μL of drug-free CAMHB, while 20 μL of QCs and test samples were mixed with 20 μL of water. 20 μL of internal standard working solution ([$^2H_5$]-piperacillin and sulbactam in water), and 100 μL of acetonitrile was added to standards, QCs and test samples, followed by vortex mixing and centrifugation for 5 minutes at 13200 ×g. A 0.5 μL aliquot of the resulting supernatant was injected onto UHPLC-MS/MS. Separation was achieved on a Shim-pack XR-ODS III (2.0 × 50 mm, 1.6 μm; Shimadzu, Kyoto, Japan) using a gradient elution of mobile phases – 0.1% formic acid (v/v); in water (A), in acetonitrile (B). Piperacillin and [$^2H_5$]-piperacillin were detected in positive ionisation mode with multiple reaction monitoring (MRM) transitions of 518.10→160.00 m/z (quantifier) and 518.10→143.15 m/z (qualifier) for piperacillin and 523.20→148.15 m/z (quantifier) and 523.20→160.05 m/z (qualifier) for [$^2H_5$]-piperacillin. Tazobactam and sulbactam were detected in negative ionisation mode with MRM transitions of 299.10→138.20 m/z (quantifier) and 299.10→68.00 m/z (qualifier) for tazobactam and 232.05→140.15 m/z (quantifier) and 232.05→188.20 m/z (qualifier) for sulbactam.

For tobramycin, 20 μL aliquots of standards were mixed with 20 μL of drug-free CAMHB, while 20 μL of QCs and test samples were mixed with 20 μL of water. 100 μL of internal standard working solution (amikacin in 2.5% w/v TCA) was added to standards, QCs and test samples, followed by vortex mixing and centrifugation for 5 minutes at 13200 ×g. A 100 μL aliquot of the supernatant was then mixed with 100 μL of acetonitrile, and a 1 μL aliquot of the resulting mixture was injected onto UHPLC-MS/MS. Separation was achieved on a Luna Omega Polar C18 column (2.1 × 50 mm, 1.6 μm; Phenomenex, USA) using a gradient elution of mobile phases - 0.2% formic acid (v/v) in 10 mM ammonium acetate; in water (A), in 90% acetonitrile (B). Tobramycin and amikacin were detected in positive ionisation mode with multiple reaction monitoring (MRM) transitions of 468.20 → 163.15 m/z (quantifier) and 468.20 → 324.15 m/z (qualifier) for tobramycin and 586.25 → 163.25 m/z (quantifier) and 586.25 → 68.60 m/z (qualifier) for amikacin.

The test samples were assayed in batches alongside calibrators and QCs and results were subject to batch acceptance criteria (1). The precision and accuracy of the assays were within acceptable limits. The precision for piperacillin was within 7.7% and accuracy was within 6.5%, based on QC concentrations of 1.5, 15, 50, and 400 mg/L. For tazobactam, precision was within 13.7% and accuracy was within 3.5%, using QC concentrations of 0.1875, 1.875, 6.25, and 50 mg/L Tobramycin assay method demonstrated precision within 8.8% and accuracy within 3.8%, based on the QC concentration of 0.6, 1.5, 15, and 40 mg/L.

DNA Extraction, Whole Genome Sequencing, and Bioinformatic Analysis.

Whole genome sequencing was conducted for the *P. aeruginosa* isolates prior to antibiotic exposure and following exposure to treatment in the HFIM for any bacterial populations that regrew after initial bacterial load reduction despite antibiotic treatment.

DNA extraction from *P. aeruginosa* samples was carried out using the DNeasy® UltraClean® Microbial Kit (QIAGEN, Venlo, Netherlands) in accordance with manufacturer's instructions. The quantity of DNA extracted was quantified using Qubit™ 1X dsDNA High Sensitivity assay kit with a Qubit™ 4.0 fluorometer (Invitrogen™, California, USA).

DNA libraries were prepared using Nextera DNA Flex Library Preparation Kit (Illumina # 20018705) following the manufacturer's protocol at Australian Centre for Ecogenomics. Nextera DNA Flex libraries were pooled at equimolar amounts of 2 nM per library to create a sequencing pool. The library pool was quantified in triplicates using the Qubit™ dsDNA HS Assay Kit (Invitrogen™, California, USA). The quality of the library was assessed using the Agilent D1000 HS tapes (#5067-5582) on the TapeStation 4200 # G2991AA (Agilent, Santa Clara, USA,) as per the manufacturer's protocol. The library was sequenced on an Illumina Nextseq2000 with XLEAP-SBS chemistry, using a 300-cycle paired end P3 flow cell, according to the manufacturer's protocol.

The raw Illumina sequencing reads were processed and analysed using a custom, inhouse microbial genomic analysis pipeline**,** SnapperRocks (2)**.** First, the raw reads were quality-trimmed using Trimmomatic v0.39 (3), removing both the low-quality bases (Q<20) and the adapter sequences. Reads shorter than 50 bp after trimming were discarded. Second, the quality of trimmed reads was assessed using FastQC version 0.11.9 (4).The quality trimmed reads were assembled using SPAdes version 3.11.1 (5). Third, assembled contigs were filtered to retain only those with a minimum coverage of 10^{x} and a minimum length of 100 bp using a custom Perl script. In silico multilocus sequence typing (MLST) was performed using MLST version 2.32.2 tool (https://github.com/tseemann/mlst) against the filtered contigs and SRST2 (6) against the reads. Finally, antimicrobial resistance (AMR) genotyping was performed using AMRFinderPlus version 4.0.19 (7), which screens for resistance genes, point mutations, and virulence factors of the isolates against the reference NCBI AMR sequence catalogue.

References

1. U.S. Food and Drug Administration. *Bioanalytical Method Validation Guidance for Industry*; U.S. Food and Drug Administration: *Silver Spring, MD, USA*, 2018.
2. Forde, B.M.; Bergh, H.; Cuddihy, T.; Hajkowicz, K.; Hurst, T.; Playford, E.G.; Henderson, B.C.; Runnegar, N.; Clark, J.; Jennison, A.V.; et al. Clinical Implementation of Routine Whole-genome Sequencing for Hospital Infection Control of Multi-drug Resistant Pathogens. *Clin. Infect. Dis*. **2023**, *76*, e1277–e1284. https://doi.org/10.1093/cid/ciac726.
3. Bolger, A.M.; Lohse, M.; Usadel, B. Trimmomatic: A flexible trimmer for Illumina sequence data. Bioinformatics **2014**, *30*, 2114–2120. https://doi.org/10.1093/bioinformatics/btu170.
4. FastQC: A Quality Control Tool for High Throughput Sequence Data. Avaliable online: https://www.bioinformatics.babraham.ac.uk/projects/fastqc/ (accessed on date).
5. Bankevich, A., Nurk, S., Antipov, D., Gurevich, A.A., Dvorkin, M., Kulikov, A.S., Lesin, V.M., Nikolenko, S.I., Pham, S., Prjibelski, A.D., et.al. SPAdes: A new genome assembly algorithm and its applications to single-cell sequencing. J Comput Biol **2012**, *19*, 455–477. https://doi.org/10.1089/cmb.2012.0021.
6. Inouye, M.; Dashnow, H.; Raven, L.-A.; Schultz, M.B.; Pope, B.J.; Tomita, T.; Zobel, J.; Holt, K.E. SRST2: Rapid genomic surveillance for public health and hospital microbiology labs. Genome Med. **2014**, *6*, 90, doi:10.1186/s13073-014-0090-6.
7. Feldgarden, M.; Brover, V.; Gonzalez-Escalona, N.; Frye, J.G.; Haendiges, J.; Haft, D.H.; Hoffmann, M.; Pettengill, J.B.; Prasad, A.B.; Tillman, G.E.; et al. AMRFinderPlus and the Reference Gene Catalog facilitate examination of the genomic links among antimicrobial resistance, stress response, and virulence. Sci. Rep. **2021**, *11*, 12728, https://doi.org/10.1038/s41598-021-91456-0.
